# Selective COX-2 Inhibitors: Road from Success to Controversy and the Quest for Repurposing

**DOI:** 10.3390/ph15070827

**Published:** 2022-07-03

**Authors:** Afaf A. El-Malah, Magdy M. Gineinah, Pran Kishore Deb, Ahdab N. Khayyat, Monika Bansal, Katharigatta N. Venugopala, Anfal S. Aljahdali

**Affiliations:** 1 Pharmaceutical Chemistry Department, Faculty of Pharmacy, King Abdulaziz University, Jeddah 21589, Saudi Arabia; aalmallah@kau.edu.sa (A.A.E.-M.); mgineinah@kau.edu.sa (M.M.G.); ankhayyat@kau.edu.sa (A.N.K.); ashaljahdali@kau.edu.sa (A.S.A.); 2 Department of Pharmaceutical Sciences, Faculty of Pharmacy, Philadelphia University, Amman 19392, Jordan; 3 Department of Neuroscience Technology, College of Applied Medical Sciences in Jubail, Imam Abdul Rahman Bin Faisal University, Dammam 31441, Saudi Arabia; mbbanasl@iau.edu.sa; 4 Department of Pharmaceutical Sciences, College of Clinical Pharmacy, King Faisal University, Al-Ahsa 31982, Saudi Arabia; kvenugopala@kfu.edu.sa; 5 Department of Biotechnology and Food Science, Faculty of Applied Sciences, Durban University of Technology, Durban 4001, South Africa

**Keywords:** selective COX-2 inhibitors, coxibs, drug repurposing, celecoxib, valdecoxib, Vioxx, etoricoxib, rofecoxib

## Abstract

The introduction of selective COX-2 inhibitors (so-called ‘coxibs’) has demonstrated tremendous commercial success due to their claimed lower potential of serious gastrointestinal adverse effects than traditional NSAIDs. However, following the repeated questioning on safety concerns, the coxibs ‘controversial me-too’ saga increased substantially, inferring to the risk of cardiovascular complications, subsequently leading to the voluntary withdrawal of coxibs (e.g., rofecoxib and valdecoxib) from the market. For instance, the makers (Pfizer and Merck) had to allegedly settle individual claims of cardiovascular hazards from celecoxib and valdecoxib. Undoubtedly, the lessons drawn from this saga revealed the flaws in drug surveillance and regulation, and taught science to pursue a more integrated translational approach for data acquisition and interpretation, prompting science-based strategies of risk avoidance in order to sustain the value of such drugs, rather than their withdrawal. Looking forward, coxibs are now being studied for repurposing, given their possible implications in the management of a myriad of diseases, including cancer, epilepsy, psychiatric disorders, obesity, Alzheimer’s disease, and so on. This article briefly summarizes the development of COX-2 inhibitors to their market impression, followed by the controversy related to their toxicity. In addition, the events recollected in hindsight (the past lessons), the optimistic step towards drug repurposing (the present), and the potential for forthcoming success (the future) are also discussed.

## 1. Introduction

**Figure 1:**
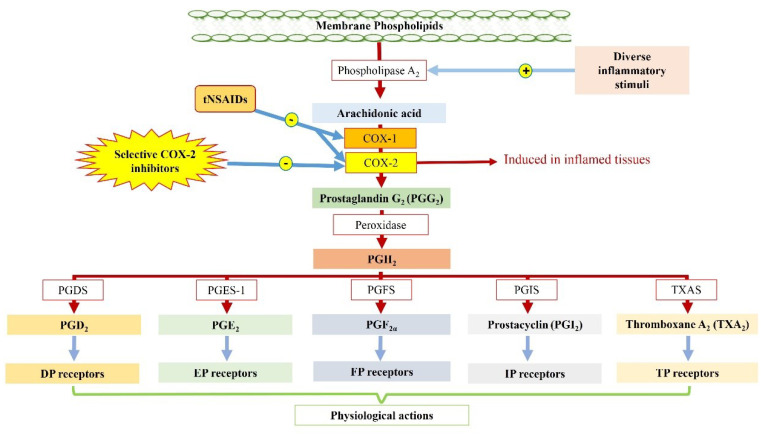
Biosynthesis of prostaglandins and associated compounds. For instance, arachidonic acid is liberated by Ca^2+^ stimulation mediated by diverse inflammatory stimuli or phosphorylation of phospholipase A_2_ from the membrane phospholipid. The cyclooxygenase enzymes (both COX-1 and COX-2) epoxygenate arachidonic acid into prostaglandin G2 (PGG_2_), which is further converted into prostaglandin H_2_ (PGH_2_) with the help of peroxidase enzyme. Following this, PGH_2_ undergoes conversion into prostaglandin (PG) analogues (i.e., PGD_2_, PGE_2_, PGF_2α_, and PGI_2_ or prostacyclin) and thromboxane A_2_ (TxA_2_) by the help of isomerases and synthases, respectively. The formed PGs and associated compounds are capable of triggering a myriad of signalling events by activating their respective membrane receptors located at the site of production. The traditional non-steroidal anti-inflammatory drugs (tNSAIDs) inhibit cyclooxygenase enzyme non-selectively and prevent prostaglandin synthesis, whereas selective COX-2 inhibitors (coxibs) inhibit the COX-2 isoform that is induced by inflammation. PGDS: prostaglandin D synthase, PGES-1: prostaglandin E synthase-1, PGFS: prostaglandin F_2α_ synthase, PGIS: prostacyclin synthase, and TXAS: thromboxane A_2_ synthase.

The family of prostaglandin-endoperoxide synthase (PTGS), commonly called as cyclooxygenase (COX), catalyses the reaction involved in the biosynthesis of prostaglandins (PGs) and associated compounds [1]. The membrane-associated COX-isoforms can trigger a sequence of reactions immediately after the release of arachidonic acid (AA) by the damaged membranes. For instance, AA undergoes biotransformation in the presence of COX to produce cyclic prostaglandin endoperoxides, viz., PGG_2_ and PGH_2_, which are further converted into prostaglandin analogues (i.e., PGD_2_, PGE_2_, PGF_2α_, and PGI_2_ or prostacyclin) and thromboxane A2 (TxA_2_) by isomerases and synthases, respectively (Figure 1) [2]. PGs are capable of triggering a myriad of signalling events by activating their respective membrane receptors located at the site of production [3]. Though COX was initially thought to be constitutively expressed in the tissues, the elevated production of PGs during inflammation and subsequent reduction after corticosteroid administration gave clues to, and later led to, the identification of an inducible isoform of the enzyme, i.e., COX-2 [4,5]. Given the difference in structure, expression, and function of these two isoforms (i.e., COX-1 and COX-2), this discovery further clarified the dual role of PGs in different physiological functions and pathological states [6]. It was hypothesized that COX-1, being a housekeeping enzyme, mediates cytoprotective action, including gastric mucosa production and the regulation of renal and platelet activity, whereas COX-2 has been found to be associated with inflammatory processes. Interestingly, the endeavour in the development of selective COX-2 inhibitors (so-called “coxibs”) over traditional non-steroidal anti-inflammatory drugs (tNSAIDs) is also attributed to the aim to overcome the frequent adverse events (e.g., gastrointestinal complications) related to the use of tNSAIDs and the non-selective inhibition of COX-1-derived protective phenomena [7,8]. Despite early questioning over the role of COX-2 in the regulation of mucosal inflammation and ulcer healing, the quest for developing new coxibs kept on gaining momentum based on the epidemiological data that demonstrated reduction in incidence of tNSAID-related adverse events. As anticipated, coxibs started to obtain marketing approval following the results obtained in relatively small and short-term human trials, and within a span of years, they had seen a huge commercial success, taking over a substantial share in the NSAIDs market. Nevertheless, the fatal flaws in the simplicity of the COX-2 hypothesis soon became apparent, followed by the teetering between drug-associated health benefits and risks. Evidently, this resulted in labelling these drugs with black box-warnings for potential cardiovascular and gastrointestinal complications, and even the voluntary withdrawal of a few coxibs from the market. Optimistically, at the time of dismay, the enthusiasm of the researchers in the quest of drug repurposing reflected the good zeal of science, which remains a major interest of this article.

## 2. Purposeful Development of Coxibs and Their Success: The COX-2 Saga

While working for Bayer Pharmaceuticals, Felix Hoffman recognized aspirin (acetyl salicylic acid) to be an excellent analgesic agent. Since then, aspirin remained the mainstay (though later repurposed) of tNSAIDs and dominated the market for over a century [9]. Subsequently, several aspirin-like drugs were developed following the success of aspirin and, most importantly, were developed either with minimal pharmacological and toxicological evaluation or after direct human trials. Examples include phenacetin, phenylbutazone, oxyphenylbutazone, indomethacin, diclofenac, sulindac, etc. [10]. By 1938, evidence regarding the gastrotoxicity associated with the chronic use of NSAIDs was distinct. Even after that, NSAIDs developed until the 1960s failed to project “a safer NSAID”. The clinical development of NSAIDs was revolutionized following the discovery and successive studies supporting that aspirin (or aspirin-like drugs) acts by inhibiting the enzyme COX, involved in the biosynthesis of PGs [11,12,13].

However, several queries remained unanswered for years, with the simple depiction of COX being involved in inflammation: (a) delayed PG generation following agonist stimulation, (b) polyoma virus-induced elevated PG synthesis in kidney fibroblasts of baby hamsters, (c) steroid-mediated differential inhibition of basal as well as induced PG production, and (d) variation in the IC50 of NSAIDs for the inhibition of PG production. Later, complementary efforts from different research groups resulted in the identification of two COX mRNAs, one (i.e., COX-1) that expresses constitutively and remains unaffected by the treatment of corticosteroids, while the other (i.e., COX-2) is induced by the inflammatory stimuli or cytokines that can be inhibited by the administration of corticosteroids [14,15,16]. This led to the development of the “COX-2 hypothesis” that suggested COX-2 inhibition to be the sole mechanism of NSAID-mediated anti-inflammatory effects, whereas COX-1 inhibition was suggested to be associated with the undesired gastrotoxicity, as supported by the presence of COX-1 in the GIT. Consistent with the fact that the existing NSAIDs non-selectively targeted COX isoforms, this hypothesis gained wide acceptance and popularity, and led to the prediction that selective COX-2 inhibition would fulfil the lacunae in the search of ‘safer NSAIDs’. Experiments with selective COX-2 inhibitors such as SC-58125 and NS-398 demonstrated inhibition of carrageenan-induced PG production, while showing no change in basal gastric PG production or any sign of gastric lesions [17]. In addition to that, extensive studies suggested that two aromatic rings connected with carbocycle or heterocycle, one substituted with sulfone or sulphonamide on *para*-position, determine the COX-2 selectivity [18,19]. For instance, celecoxib and valdecoxib possess a sulphonamide-substituted pyrazole ring and oxazole ring, respectively, while rofecoxib and etoricoxib consist of a sulfone-substituted furanone ring and pyridine ring, respectively.

Subsequently, many pharmaceutical companies pursued efforts to develop coxibs, based on the rationale that selective COX-1 sparing would improve GI safety [20]. Reports from the outcome trials, CLASS [21], VIGOR [22], and TARGET [23], including 9000 to 18,000 subjects with rheumatoid arthritis or osteoarthritis, demonstrated reduction in haemorrhagic GI complications by half or two-thirds with coxibs as compared with naproxen or ibuprofen (these trials are discussed in the next subsection). Nevertheless, the two coxibs, viz., celecoxib and rofecoxib, convincingly demonstrated their advantages over tNSAIDs. Celecoxib (**1**; 4-[5-(4-methylphenyl)-3-(trifluoromethyl)pyrazol-1-yl]benzenesulfonamide) was first launched in February 1999, representing one of the rapid drug development efforts in pharmaceutical history. The grandiose launch of celecoxib was marked by sales of about 1.5 billion USD in the first year. The FDA recommended celecoxib, along with other NSAIDs, such as acetaminophen, as a first-line therapy for rheumatoid arthritis (RA) and osteoarthritis (OA). Celecoxib is also used for the management of primary dysmenorrhoea and acute pain in women and as an adjunct therapeutic option for familial adenomatous polyposis [24]. Following the success of celecoxib, rofecoxib (**2**; 3-(4-methylsulfonylphenyl)-4-phenyl-2H-furan-5-one) was launched after six months and garnered around 400 million USD in the initial year [10]. The aggression in the marketing and advertising was quite remarkable, i.e., rofecoxib advertising expenditures were the highest in 2000, while celecoxib ranked seventh among all the commercially available drugs [25]. In addition, substantial direct-to-consumer advertising led to exponential market turnover along with the development of a general perception of safer drugs among the common people. In November 2001, a second-generation coxib, viz., valdecoxib (**3**; 4-(5-methyl-3-phenyl-1,2-oxazol-4-yl)benzenesulfonamide), was approved by the Food and Drug Administration (FDA) for the management of OA and RA, which also remained a blockbuster coxib until 2005. Ultimately, the period from 1999 to 2005 was marked as “the COXIB-era”, with an expeditious rise and an even more rapid downfall.

## 3. Controversy in the COX-2 Saga: The Downfall

Based on the COX-2 hypothesis, the developed coxibs were tested in several clinical trials for their effectiveness in patients with RA. The Vioxx Gastrointestinal Outcomes Research (VIGOR) trial conducted by Merck was one such study, carried out to address the effectiveness of rofecoxib (Vioxx) and its impact on GI toxicity (a major drawback of tNSAIDs) in patients. This trial compared rofecoxib (with a 50 mg single daily dose) and naproxen (with a 500 mg twice daily dose) in 8076 RA patients. The VIGOR trial revealed a similar efficacy of both drugs against RA, and it demonstrated reduction by approximately half the incidence of serious GI lesions with the long-term use of rofecoxib compared to naproxen. Unexpectedly, the rofecoxib group was associated with a higher incidence of myocardial infarction compared to the naproxen group. Although the absence of a placebo arm in the trial made the results inconclusive, the authors of the study speculated naproxen to be cardioprotective (like aspirin), an effect probably mediated via the inhibition of TxA_2_ synthesis [22]. Controversy loomed as the data based on which rofecoxib was granted approval were not published in peer reviewed journals for about one and a half years (until November 2000) after commercial approval. Additionally, it remains uncertain why the Arthritis Advisory Committee of the FDA took almost two years to conduct the first meeting to discuss the potential cardiovascular hazards associated with rofecoxib use. Furthermore, the FDA’s decision making had been questioned over time for not mandating any prospective trial to assess the cardiovascular risks associated with rofecoxib use and for not stopping Merck from using direct-to-consumer advertisements, on which Merck was spending around 100 million USD per year. Merck also allegedly published a series of literatures to reinforce the message among the general audience that rofecoxib possesses no cardiovascular risks; rather, it was emphasized that naproxen was cardioprotective [26,27]. Meanwhile, in April 2002, the FDA instructed the company to include precautionary labels about the potential cardiovascular risks. Since January 2002 to August 2004, several epidemiological studies implied the potential risk of cardiovascular hazards from Vioxx [27,28,29]. A conclusion to the dilemma came only by happenstance, in the Adenomatous Polyp Prevention on Vioxx (APPROVe) study that discovered an increased risk of myocardial infarction and stroke in rofecoxib-treated patients, leading to the premature cessation of the study [30]. The blockbuster status of coxibs was dubious, as there were several epidemiologic studies suggesting the risk of cardiovascular events associated with rofecoxib use, but Merck, the producer, claimed all these studies to be flawed and affirmed randomized clinical trials to be suitable for determining the risks, which were never initiated. Keeping everyone in awe, in September 2004, Merck decided to voluntarily withdraw Vioxx, or rofecoxib, from the market. Immediately after this withdrawal, attention turned to celecoxib (Celebrex) and valdecoxib (Bextra), which were projected to be profitable at that time. Very soon afterward, in the Coronary Artery Bypass Surgery (CABG-II) study, it was reported that valdecoxib and its prodrug (parecoxib) were associated with an elevated risk of cardiovascular events [31,32], and the “me-too saga” continued with the early discontinuation of the Adenoma Prevention with Celecoxib (APC) trial, reporting cardiovascular hazards and subsequent deaths [33]. After these events, the FDA convened a joint meeting in February 2005 that amended label warnings, containing potential cardiovascular and GI risks, [34] for both coxibs and tNSAIDs. Additionally, the committee recommended Pfizer to voluntarily withdraw valdecoxib, as the overall risk/benefit profile was unfavourable. The European Medicines Agency (EMEA) also asked Pfizer to pull the drug from the European market [35]. These subsequent decisions questioned the drug regulatory system on drug approval. It is estimated that more than 27,000 lawsuits related to the cardiovascular hazards associated with the use of Vioxx were filed against Merck. Even after the continuous denying of the fact that Merck withheld certain material information from the FDA during the fast-track process, the company decided to defend each and every Vioxx litigation. Although Merck did not admit any fault or causation, on November 2007, the pharma giant ‘blinked’ and agreed to settle the Vioxx litigation for 4.85 billion USD [34]. Pfizer also reached a litigation settlement at 486 million USD for the accusation that Celebrex and Bextra caused health issues [36]. This highlighted the apparent blind spot in the drug approval and marketing process, ultimately raising serious concerns, including patient harm, physician apprehension, the filing of lawsuits, and even declining faith in the already approved drugs. Nevertheless, the recent expeditious pace as seen in the coronavirus disease 2019 (COVID-19) drug development also remains dubious from this vantage point, as observed from the mixed response among the scientific communities and general population. As a consequence of the class effect, the Vioxx successor, etoricoxib (Arcoxia)—a second-generation coxib—was also rejected by the FDA for its approval. The same was the case for lumiracoxib (Prexige), another second-generation coxib that was not approved in the US and later withdrawn from the market in other countries due to serious liver toxicity [37,38,39].

## 4. The Quest of Repurposing and Efforts from Medicinal Chemists

Now that the controversy has settled, the availability of coxibs varies by country, with prescribing restrictions and variable sales suspensions [40]. Nevertheless, one must accept the fact that no drug comes without side effects. Therefore, optimistically, researchers cannot afford to lose the hardship used to bring a drug onto the market. As repurposing a drug for novel therapeutic indications apart from the conditions for which the drug was originally approved capitalizes on prior investments while using the existing efficacy and safety data based on the clinical trials conducted, it can bring about a new utility of the dethroned COX-2 inhibitors. Nevertheless, coxib repurposing is anticipated to resurrect the drugs back onto the market. The following subsections describe the efforts laid down to find the potential of individual coxibs against various conditions. A list of clinical trials currently recruiting for the evaluation of coxibs in repurposing for different disease conditions are provided in Table 1.

### 4.1. Celecoxib (Celebrex)

**Figure 2:**
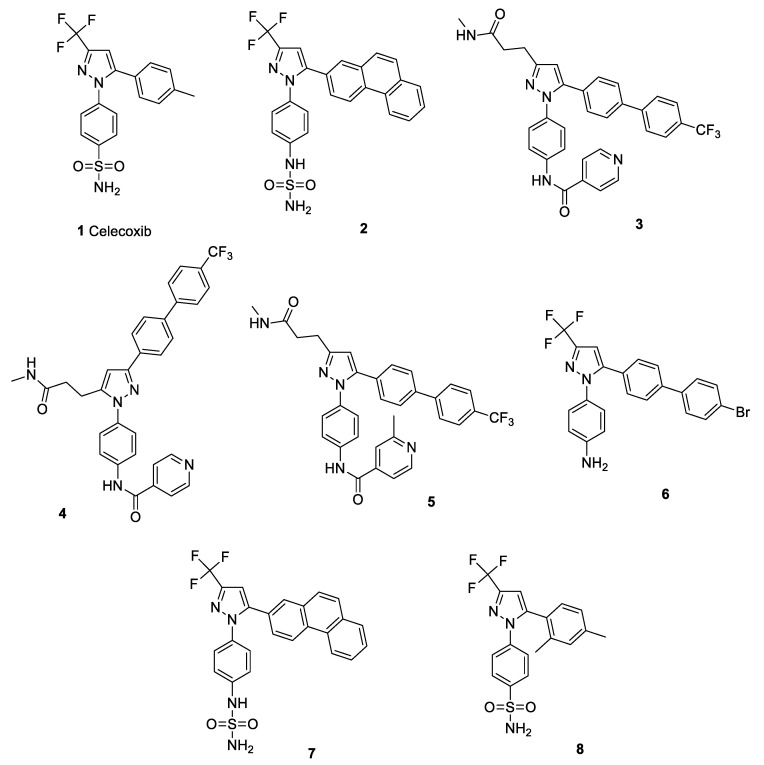
Structure of celecoxib (**1**) and its potent derivatives (**2**–**8**).

Celecoxib (**1**) is currently available on the market as a prescription medication in the form of capsules in 50, 100, 200, and 400 mg doses, containing boxed warnings of CV hazards. Several studies have demonstrated a causal link between the expression of the multi-drug resistance 1 (MDR1) gene or P-glycoprotein (involved in the resistance towards cancer chemotherapeutics and antimicrobial agents) and COX-2. It was reported that COX-2 controls MDR1 expression in malignancy; thus, drug resistance can be reversed by the use of celecoxib [41,42,43,44]. Celecoxib (at 6.25 μM and 12.5 μM) was reported to sensitize methicillin-resistant *Staphylococcus aureus* (MRSA) and *Mycobacterium smegmatis* for ampicillin, kanamycin, chloramphenicol, and ciprofloxacin by enhancing drug accumulation via the inhibition of the MDR efflux pump [45]. Supporting this fact, another study reported that celecoxib affects the bacterial membrane potential and cellular permeability, particularly via interfering with the Na^+^/K^+^ ion transporter, and thereby enhancing the ampicillin uptake by *S. aureus* [46]. A recent study reported that a celecoxib derivative (**2**) is capable of eradicating resistant *S. aureus* (including MRSA) and its biofilms via the inhibition of YidC2 translocase [47]. A previous study suggested that celecoxib possesses broad-spectrum activity against Gram-positive bacteria, including *Staphylococcus* spp., *Streptococcus* spp., *Listeria* spp., *Bacillus* spp., and *Mycobacterium* spp., but not against Gram-negative bacteria. The possible mechanism of action involves the dose-dependent prevention of replication, transcription, and protein translation. Topical celecoxib application (1–2%) significantly decreased mean bacterial count in an MRSA-infected mouse model, along with a reduction in inflammatory cytokines (e.g., tumour necrosis factor-α, interleukin-1 beta, interleukin-6, and monocyte chemo attractant protein-1) in MRSA-infected wounds [48]. A series of celecoxib analogues, compounds **3**, **4,** and **5,** demonstrated potent inhibitory activity against *M. tuberculosis* (including MDR strains) and *S. aureus* [49]. Contrary to this, another study reported no bactericidal activity from celecoxib (alone or in combination with rifampicin or pyrazinamide) against *M. tuberculosis* in a whole-blood assay, suggesting no involvement of eicosanoid pathways and efflux pumps in mycobacterial growth [50]. Celecoxib-based compound **6** was also reported to exhibit inhibitory activity against type A *Francisella tularensis* (SchuS; a virulent strain), the live vaccine strain (LVS; type B) of *F. tularensis*, and *F. novicida* (an avirulent strain) in a growth medium [51]. However, this bactericidal action was not seen in the case of rofecoxib. Similar findings were reported for compound **7**, a celecoxib derivative that exhibited inhibitory activity against SchuS4 and LVS, without any significant host toxicity. In a tularaemia mouse model, compound **7** prolonged survival duration from a lethal dose of SchuS4 and prevented 50% of the population from lethal LVS infection [52]. Recently, a celecoxib analogue (**8**) (Figure 2) was reported to be active against *Acinetobacter baumannii* (MIC = 4 μg/mL), *S. aureus* (MIC = 4 μg/mL), and MRSA isolates (MIC = 4–16 μg/mL) when combined with colistin B. It also exhibited mild improvement in the survival of MRSA-infected mice and was found to possess inhibitory activity against *S. aureus* DNA gyrase (122.8 μg/mL) and dihydrofolate reductase (105.1 μg/mL) [53]. Celecoxib was also reported to increase the efficacy of low-dose imipenem and ampicillin against polymicrobial sepsis in mice and ESKAPE pathogens, respectively [54]. Structure of celecoxib and its potent derivatives (as discussed above) are shown in Figure 2.

Interestingly, positive correlation of elevated urine PGE_2_ levels with that of COVID-19 progression led to the evaluation of Celebrex (celecoxib) in clinical trials for its potential against severe acute respiratory syndrome coronavirus 2 (SARS-CoV-2) [55,56]. A prospective clinical trial (ChiCTR2000031630) reported Celebrex (as adjuvant therapy) to promote recovery from all COVID-19 types and to reduce the mortality among aged patients and those with comorbid conditions [57]

Celecoxib was approved by the FDA in 1999 for familial adenomatous polyposis [58]. Celecoxib is reported to induce apoptosis [59] and immune cell-regulated tumour cell lysis [60] and to inhibit the progression of cell cycle [61], metastasis, and angiogenesis [62]. In addition, increased COX-2 expression was found to be associated with different types of malignancies, including breast cancer, liver cancer, gastric cancer, and oesophageal cancer. Given the potential of inhibiting the mechanisms involved in cancer progression, celecoxib was evaluated in several clinical trials for different tumours. Metronomic chemotherapy with celecoxib (200 mg taken orally twice daily) and cyclophosphamide (50 mg taken orally daily) was reported to demonstrate an overall benefit rate of 46.7% in advanced breast cancer patients [63]. A similar dose of celecoxib (combined with carboplatin) also exhibited promising activity in recurrent heavily treated ovarian cancer [64]. In contrast to these, celecoxib, in combination with isotretinoin and thalidomide to dose-dense temozolomide, did not establish any significant benefit in newly diagnosed patients with glioblastoma [65]. A meta-analysis revealed that celecoxib, in combination with chemotherapeutic agents, improves the overall response rate in non-small-cell lung cancer (NSCLC) [66]. A recent preclinical study also showed that a combination of celecoxib and metformin can prevent the occurrence of hepatocellular carcinoma by causing higher cytotoxicity in the cancerous cells [67]. Celecoxib was earlier reported to be a powerful tool to enhance dendritic cell-based immunotherapy [68]. Though there exist abundant clinical trials suggesting the potential of celecoxib against cancer, the responsiveness of different populations needs to be investigated to ascertain its suitability.

As inflammatory conditions with increased levels of prostaglandins and pro-inflammatory cytokines are associated with the etiopathogenesis of major depression, celecoxib has been studied in clinical trials for its effectiveness in depression. A double-blind prospective study (*n* = 40) reported celecoxib to cause significant improvement in depressive symptomatology as compared to the reboxetine group [69]. An Iranian trial reported celecoxib to enhance the onset of action of sertraline and resulted in a higher remission rate in major depressive patients [70]. A meta-analysis of randomized clinical trials reported celecoxib to decrease depressive symptoms with reduced adverse effects; however, the mean estimate was claimed to be uncertain due to a high risk of bias and heterogeneity [71]. An exploratory study revealed that celecoxib augmentation of anxiolytic or antidepressant drugs exhibits positive effects on anxiety, depression, and mental well-being [72]. In contrast to these findings, recently published data of a placebo-controlled, double-blind, randomized trial reported that celecoxib augmentation of vortioxetine has no evidence of superior efficacy as compared to a placebo in mitigating depressive severity [73].

COX-2-mediated inflammation is known to be involved in the development of insulin resistance associated with type 2 diabetes mellitus (T2DM) and obesity [74,75,76,77]. An Egyptian study suggested that celecoxib administered with glimepiride prevents glycemia, insulin resistance, and obesity-associated inflammation [78]. Celecoxib is also known to ameliorate non-alcoholic steatohepatitis in T2DM via the suppression of non-canonical Wnt5a/JNK1 pathways [79]. Another finding showed celecoxib to down-regulate the hippocampal COX-2 expression and up-regulate the BDNF-TrkB pathway to reverse memory deficits in a diabetic rat model [80]. In addition to that, celecoxib was found to ameliorate diabetic neuropathy via the inhibition of oxidative stress and apoptosis through the modulation of miR-155 in dorsal root ganglion neurons [81].

The implications of neuroinflammation in Parkinsonism led to the evaluation of celecoxib for its protective effect in the progression of the disease. A preclinical study reported celecoxib to prevent the progressive degeneration of dopaminergic cells via the inhibition of microglia activation in a 6-hydroxydopamine (6-OHDA) model [82]. Similarly, treatment with celecoxib attenuated a lipopolysaccharide (LPS)-induced increase in activated microglia and astrocyte levels, and led to a significant improvement in dopaminergic dysfunction [83]. It was also reported that celecoxib is capable of increasing viable CA1 pyramidal neurons by decreasing neuronal apoptosis [84]. A recent study found that celecoxib up-regulates the expression of neuroprotective markers, including apolipoprotein D, transcription factor B, and microphthalmia-associated transcription factor in the 6-OHDA and paraquat models of Parkinsonism [85]. However, no clinical trials have been registered yet. Several speculations were made based on the epidemiological data regarding the effectiveness of celecoxib in the treatment of Alzheimer’s disease [86]. Nevertheless, results from the randomized clinical trials dissipated hope by revealing no significant preventive ability of the drug [87,88].

### 4.2. Rofecoxib (Vioxx)

**Figure 3:**
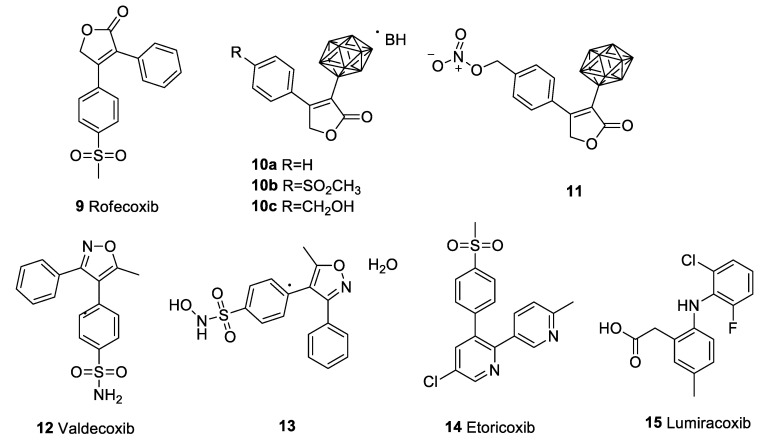
Structure of rofecoxib (**9**) and its derivatives (**10a–c**,**11**), valdecoxib (**12**) and its derivative (**13**), etoricoxib (**14**), and lumiracoxib (**15**).

Rofecoxib (**9**) was approved for the treatment of arthritis, acute pain, and menstrual pains. It was withdrawn from the market by Merck in 2004. Though a lot of experiments have been conducted to evaluate the potential of celecoxib against a variety of pathogenic microorganisms, rofecoxib failed to show significant antimicrobial properties [89]. Similarly, several studies that aimed to assess the anticancer properties of rofecoxib gained mixed results. Rofecoxib was found to inhibit cellular proliferation and induce apoptosis dose-dependently. Additionally, rofecoxib synergistically sensitized different concentrations of anticancer agents, including 5-fluorouracil, cisplatin, and etoposide, on the gastric cancer cell lines [90]. Another study suggested a limited dose-dependent effect on cell proliferation in human colorectal carcinoma cell lines [91]. In a study, rofecoxib (40 μΜ), in combination with docetaxel, was found to be more cytotoxic to lung cancer cell lines than docetaxel alone [92]. Rofecoxib was also reported to prevent DNA damage induced by ultraviolet B radiation and copper ions. In vitro inhibition of the chelation of copper ions with DNA molecules was suggested to prevent DNA damage [93]. Combined therapy of rofecoxib with HET0016 (a 20-HETE inhibitor) reduces colon tumour growth compared to rofecoxib alone. Most interestingly, this combination reduces the cerebrovascular risks associated with rofecoxib use [94]. In contrast to that, an earlier phase III clinical study demonstrated that rofecoxib improved the quality of life and response rate; however, it failed to prolong patient survival [95]. Another randomized phase III clinical trial revealed no significant improvement in the overall survival and recurrence of colorectal cancer [96]. Efforts from medicinal chemists to produce potent rofecoxib derivatives include the synthesis of carboranyl moiety containing rofecoxib analogues, viz., compounds **10a–c** and **11** (Figure 3). These compounds were produced by replacing the phenyl group at 3-position of rofecoxib with *o*-carborane or a *nido*-carborane cluster. These compounds were cytotoxic against melanoma and colon cancer cell lines; however, the selectivity towards the COX-2 enzyme was compromised [97]. Another group reported the development of a hybrid drug (named KSS19) by combining rofecoxib with cis-stilbene from combretastatin A4, which was found to inhibit tubulin polymerization and malignant cell migration/invasion, and exhibited the activation of NF-κB/Snail pathways in colorectal cancer cell lines [98].

Based on the implications of inflammatory mechanisms in neurodegenerative disorders like Parkinsonism and Alzheimer’s disease, rofecoxib was hypothesised to be preventive in the disease progression [99]. Earlier studies showed that rofecoxib can attenuate the excitotoxic hippocampal neuronal injury and that it prevented injury-induced cerebral oedema [100]. However, a randomized, placebo-controlled, double-blind, multi-centred trial reported rofecoxib (as well as low-dose naproxen) to not be effective in the prevention of cognitive decline in mild-to-moderate Alzheimer’s patients [101]. Similar findings were also reported by another clinical trial by the same researcher [102]. A clinical trial from Iraq reported rofecoxib to significantly improve the sensory component of choice reaction time tasks, but the CNS integrative activity was insignificant [103]. A study on mild cognitive impairment (MCI) patients revealed rofecoxib does not play any significant role on the delay of Alzheimer’s disease diagnosis [104]. Contrastingly, another study drew the conclusion that rofecoxib might accelerate the pathophysiological events in Alzheimer’s disease to increase the AD diagnosis rate in MCI patients [105].

Most importantly, Tremeau Pharmaceuticals, a Massachusetts-based biotech start-up, is currently enrolling for a pivotal phase III clinical trial of rofecoxib (TRM-201) for haemophilic arthropathy, a recurring, debilitating intra-articular bleeding disorder. TRM-201, a 17.5 mg rofecoxib containing an oral dosage form, along with its PK profile, is protected by U.S. Patent No. 10945992, while highly purified rofecoxib is covered under U.S. Patent No. 10987337. Currently, Vioxx is a registered trademark of Tremeau Pharmaceuticals. As there is no FDA-approved drug on the market for haemophilic arthropathy, if found satisfactory, TRM-201 would be the first specifically indicated non-opioid drug for haemophilic arthropathy. Tremeau Pharmaceuticals is also ready with a phase III trial for assessing TRM-201 for its efficacy on migraines and primary dysmenorrhea with von Willebrand disease [106].

### 4.3. Valdecoxib (Bextra)

The FDA recommended the voluntary withdrawal of valdecoxib (Bextra; **12**) from the market based on the potential CV hazards and life-threatening skin reactions, such as toxic epidermal necrolysis, erythema multiforme, and Stevens–Johnson syndrome. Considering the quest of repurposing, a researcher group reported the synthesis of valdecoxib metabolite, N-hydroxy-4-(5-methyl-3-phenylisoxazol-4-yl)benzenesulfonamide, and its stabilization into a monohydrate form (**13**) (Figure 3), which was reported to be more potent in analgesic and anti-inflammatory activity, with a longer duration of action [107]. Another study revealed that valdecoxib and its prodrug parecoxib exhibit analgesic activity via the partial modulation of cannabinoid 1 receptors [108]. Valdecoxib was also found to inhibit PGE_2_ concentration in the ischemic penumbral cortex in a temporary focal ischemic rat model [109].

Like all other coxibs, valdecoxib was also studied for its efficacy against various cancer types. Valdecoxib treatment showed a reduction in hepatic satellite cell activation and proliferation in liver fibrosis via the down-regulation of cyclin D and cyclin E in an animal model [110]. Valdecoxib also significantly inhibited MCF-7 cell proliferation by inducing apoptosis (increased Bax expression and reduced Bcl-2 expression) and/or by increasing ROS [111].

The systemic administration of valdecoxib (Bextra) in patients with visual loss due to cystoid macular oedema showed fast and persistent improvement in vision within 10 days of therapy [112]. Contrary to this study, another study on valdecoxib on prophylactic use reported it to not influence the rate of macular oedema following scleral buckling surgery [113].

### 4.4. Etoricoxib (Arcoxia)

Although Merck & Co. applied for a new drug approval (NDA) for etoricoxib (Arcoxia, **14**) (Figure 3) for the treatment of osteoarthritis, the FDA did not approve the NDA. Etoricoxib has been studied for its potential against several conditions. Etoricoxib (10 mg/kg) exhibited reduction in incidence of colorectal cancer and growth in rats [114]. Etoricoxib was also reported to inhibit macrophage M2 polarization induced by hypoxic breast cancer cells and to suppress pro-angiogenic and the pro-invasiveness of tumour-associated macrophages [115]. Etoricoxib nanoemulsion was found to exhibit a substantial cytotoxic effect against lung cancer cell lines compared to the free drug, as well as induce apoptotic/necrotic cell death and arrested S-phase of the cell cycle [116]. Another study reported etoricoxib to suppress the dose- and time-dependent inhibition of cellular proliferation, apoptosis induction, and DNA damage in human cervical cancer cell lines [117]. An experiment suggested a combination of etoricoxib and atorvastatin to possess a chemopreventive effect against DMH-induced colon cancer [118]. Another study also reported dose-dependent antitumor activity of etoricoxib in mammary carcinogenesis [119]. A very recent study reported etoricoxib to significantly suppress alpha-fetoprotein and carbohydrate antigen 19–9, the liver tumour biomarkers. It also improved diethylnitrosamine and 2-acetylaminofluorene-induced histopathological lesions and the infiltration of inflammatory cells, thus representing itself as an anticarcinogenic agent [120]. Similar findings suggested anti-inflammatory properties of etoricoxib, correlating its efficacy to hypoxia associated with type-B aortic dissection patients [121]. Preclinical findings also suggested etoricoxib to deter the deleterious effect of metabolic syndromes [122].

Etoricoxib was also tested in an open-label, randomized phase I/II trial (NCT02503839) for the efficacy of an etoricoxib combined H56:IC31 vaccine candidate against pulmonary and extrapulmonary tuberculosis. However, this study reported no significant effect of etoricoxib [123]. Implications from neurodegeneration involved in Alzheimer’s disease led to the evaluation of etoricoxib in a colchicine-induced disease model. The administration of etoricoxib demonstrated anxiolytic activity and a reduction in corticosterone levels. This study also claimed to inhibit neuroinflammation by the suppression of COX-2-associated neurodegeneration [124]. In search of a connection between COX-2 and epileptogenesis, researchers found that both acute and early long-term treatment with etoricoxib (10 mg/kg and 20 mg/kg i.p.) significantly reduced the number and duration of spike-wave discharges in an absence seizure animal model by ~50% and ~40%, respectively, thus representing its antiepileptogenic effect [125].

A population-based nation-wide retrospective cohort study from Taiwan revealed that etoricoxib (as well as celecoxib) consumption might be associated with a decreased risk of coronary artery diseases in rheumatoid arthritis patients as compared to the non-users of coxibs [126]. A similar study suggested etoricoxib to be effective in the reduction of dementia in osteoarthritis patients [127].

It was demonstrated in a double-blind, controlled, randomized trial that the perioperative administration of a combination of etoricoxib and duloxetine diminished postoperative pain and reduced morphine consumption after lumbar laminectomy. This study showed effective reduction in opioid-related adverse effects as compared to the monotherapies with both drugs [128]. Thus, this combination was believed to be useful as an opioid adjuvant as part of a multi-modal analgesic regimen in the management of the acute postsurgical setting.

### 4.5. Lumiracoxib (Prexige)

Lumiracoxib (**15**) (Figure 3) is a second-generation coxib that failed to gain FDA approval (however, it is approved in some countries) even after a lot of speculation. Several efforts have been made to repurpose lumiracoxib in other conditions. Lumiracoxib was reported to possess an antiproliferative effect on NSCLC cell lines [129]. The elucidation of the mechanism of action of lumiracoxib on human lung cancer cells revealed the apoptotic effect to be mediated via the regulation of Bcl-2 via ERK pathway in human NSCLC cell lines [130]. The systemic administration of lumiracoxib also demonstrated partial but significant inhibition of choroidal neovascular membrane development in age-associated macular degeneration [131].

## 5. Conclusions and Future Perspectives

The remarkable period from 1999 to 2005 was marked as “the Coxib-era”, with an expeditious rise and even more rapid downfall, keeping celecoxib as the only FDA-approved drug of its kind to be available on the market. The lessons that can be drawn from the “COX-2 debacle” can be assessed in many ways. In our opinionated view, blockbuster tagging is sometimes backed by scientific plausibility, but often, it is exaggerated for publicity by the registration authorities prior to the availability of sufficient pharmacovigilance data or epidemiologic evidence—a vantage point to be reflected upon. As exemplified by the controversial COX-2 saga, drug approval and public marketing should be vigilantly re-examined, as highlighted by the deep flaws in coxibs’ approval, marketing, and post-marketing surveillance. This might be solved by the inclusion of probationary periods to gather more safety data prior to the endorsement of the unrestricted use of a new drug, without necessarily delaying the approval processes. The regulatory authorities should adopt suitable revisions that prudently improvise the drug approval and marketing process without losing the existing strengths. Importantly, addressing the lack of ethics in the conduction of scientific studies remains another serious concern. In the authors’ opinion, publishing scientifically manipulated data by prevaricating reputed journals causes additional detriment to the scientific community (as was done by Merck during the COX-2 saga by publishing a series of misleading literatures). The exemplary retraction of the Lancet [132] and NEJM [133] studies that halted the WHO Solidarity trial related to COVID-19 is a recent example of such cases. This raises questions on the practice of scientific conduct and ethics. Therefore, ethical and professional standards should be practiced by both researchers and the regulatory bodies for the benefit of humankind. Additionally, the approval of drugs based on larger randomized human trials should be assessed carefully in safety reviews in order to meet societal expectations. Nevertheless, as the longer coxib therapies are associated with detrimental cardiovascular risk, prescriptions of shorter-duration low-dose regimens with the least frequency of administration can be practiced (through evidence-based studies, of course). Optimistically, science must move on; considering the “Coxib controversy” as a learning curve, several researchers have looked forward to the repurposing of these drugs. Of several efforts discussed in this article, Tremeau Pharmaceuticals opting for repurposing Vioxx (rofecoxib) by creating a window of opportunity to develop it as a non-opioid treatment for haemophilic arthropathy as a “niche treatment segment”, where the benefits supersede the cardiovascular risks, is an appealing strategy. Hopefully, looking into the efforts from scientists, the world can expect coxibs as repurposed drugs for niche patient segments in the near future. In addition, the attainment of higher COX-2 selectivity might open up avenues for newer scaffolds with desired chemical and pharmacological features without, or with minimal, untoward effects. Apart from these, considering all coxibs as “traitorous Vioxx offspring” should not be practiced in science, as some of these analogues may be the future blockbuster. Nonetheless, the conjugation of coxibs to nanocarriers, which was arguably never emphasized in the COX-2 saga, merits more research interest, particularly to assess the possibility of overcoming coxib-associated cardiovascular risks using nanotechnology. Further in-depth studies and incorporation of advanced scientific tools, such as molecular hybridization techniques, are anticipated to glorify the next generation of selective COX-2 inhibitors onto the market.

## Figures and Tables

**Table 1:** A list of currently recruiting clinical trials including celecoxib, rofecoxib, and etoricoxib in the intervention arm.

Intervention (Drug)	CT Phase and Identifier	Sponsor(s)	Objective of the Study
Celecoxib	Phase 4 NCT04814355	Stony Brook University/Brain & Behavior Research Foundation	To assess the effect of celecoxib on neuroinflammation associated with major depressive disorder
	Phase 2 NCT03896113	Cliniques universitaires Saint-Luc- Université Catholique de Louvai	To evaluate the influence of prior administration of celecoxib in endometrial cancer
	Phase 1 NCT04120636	Targeted Therapy Technologies, LLC	To determine the effect of sequestered transscleral celecoxib delivery in macular oedema and inflammatory eye disorders
	Phase 2 NCT04673578	University of British Columbia	To assess the efficacy of adjunctive celecoxib to treatment-as-usual in obsessive-compulsive disorder
	Phase 4 NCT04147013	Lawson Health Research Institute	To determine the effect of celecoxib on postoperative narcotic use, aspirin-exacerbated respiratory disease (AERD), and chronic rhinosinusitis
	Phase 4 NCT03645187	Tanta University	To evaluate the efficacy of adjunctive celecoxib therapy to cancer chemotherapy in metastatic colorectal cancer patients
	Phase 2 NCT03498326	Zhejiang University	To determine the efficacy of a combination of celecoxib and gemcitabine in the treatment of R0 resection pancreatic cancer
	Phase 2 NCT04786548	New York State Psychiatric Institute	To examine the effectiveness of celecoxib in combination with ongoing medication in the treatment of obsessive-compulsive disorder (OCD)
	Phase 2 NCT04162873	University of Utah	To assess the efficacy of celecoxib adjunct to standard-of-care therapy in the treatment of patients with advanced head and neck cancer
	Phase 1 NCT02885974	Baylor College of Medicine	To determine the efficacy of celecoxib combined with cisplatin and gemcitabine in the neoadjuvant treatment of localized muscle-invasive bladder cancer
	Phase 2 NCT04093323	Roswell Park Cancer Institute	To study the combination of the polarized dendritic cell (aDC1) vaccine, celecoxib, interferon α-2, and rintatolimod in the treatment of patients with refractory HLA-A2(+) melanoma.
	Phase 1/2 NCT03686657	ARKAY Therapeutics	To determine the effect of RK-01 co-administered with celecoxib, valsartan, and metformin-HCl XR on insulin resistance
	Phase 1/2 NCT03926338	Sun Yat-sen University	To determine the effect of celecoxib combined with anti-PD-1 monoclonal antibody (mAb) in the treatment of dMMR/MSI-H phenotype resectable colorectal cancer
	Phase 2 NCT03026140	The Netherlands Cancer Institute	To assess the effectiveness of celecoxib, nivolumab, and ipilimumab in early-stage colon cancer
	Phase 1 NCT04081389	Roswell Park Cancer Institute	To determine the effect of chemokine modulation therapy (including celecoxib, recombinant interferon α-2, and rintatolimod) and standard chemotherapy administered prior to surgery in treating subjects with early-stage triple-negative breast cancer
	Phase 2 NCT01356290	Medical University of Vienna	To assess the effect of biweekly bevacizumab (i.v.) in combination with celecoxib, thalidomide, fenofibrate, etoposide, and cyclophosphamide in the treatment of recurrent, progressive medulloblastoma, and ependymoma
	Phase 2/3 NCT00268476	Medical Research Council	To assess the multiple therapeutic strategies (including a celecoxib arm) in the treatment of metastatic hormone-naïve prostate cancer
Rofecoxib	Phase 3 NCT04684511	Tremeau Pharmaceuticals	To determine the safety and efficacy of rofecoxib (TRM-201) in subjects with haemophilic arthropathy
Etoricoxib	Phase 1 NCT04830579	Pharmtechnology LLC	To determine the bioequivalence of two formulations of etoricoxib by Pharmtechnology and Merck
	Early Phase 1 NCT05142098	Dow University of Health Sciences	To compare the anti-inflammatory effect of etoricoxib and pre-emptive dexamethasone following impacted third molar surgery
	Phase 3 NCT04968158	Laboratorios Silanes S.A. de C.V.	To compare and determine the safety and efficacy of a combination of etoricoxib and tramadol compared with a combination of acetaminophen and tramadol in the treatment of acute low back pain

## Data Availability

Data sharing not applicable.

## Abbreviations

6-OHDA	6-Hydroxydopamine
AA	Arachidonic acid
APC	Adenoma Prevention with Celecoxib
APPROVe	Adenomatous Polyp Prevention on Vioxx
CABG-II	Coronary Artery Bypass Surgery
COVID-19	Coronavirus disease 2019
COX	Cyclooxygenase
EMEA	European Medicines Agency
MCI	Mild cognitive impairment
MDR1	Multi-drug resistance 1
MRSA	Methicillin-resistant *Staphylococcus aureus*
NSCLC	Non-small-cell lung cancer
OA	Osteoarthritis
PG	Prostaglandin
PTGS	Prostaglandin-endoperoxide synthase
RA	Rheumatoid arthritis
T2DM	Type 2 diabetes mellitus
tNSAIDs	Traditional non-steroidal anti-inflammatory drugs
TxA_2_	Thromboxane A2
VIGOR	Vioxx Gastrointestinal Outcomes Research
